# Human milk-derived extracellular vesicles promote the heat shock response in polarized microglia

**DOI:** 10.1016/j.cstres.2025.100088

**Published:** 2025-06-26

**Authors:** Jasmyne A. Storm, Jueqin Lu, Mon Francis Obtial, Sanoji Wijenayake

**Affiliations:** Department of Biology, Richardson College for the Environment and Science Complex, The University of Winnipeg, Winnipeg, Manitoba, Canada

**Keywords:** Human microglia (HMC3), Interferon-γ (IFN-γ), Milk-derived extracellular vesicles (MEVs), Heat shock response (HSR), Cytoprotection

## Abstract

Milk-derived extracellular vesicles (MEVs) combat acute and chronic pro-inflammation in peripheral cells and tissues. However, the biological functions of MEVs in the central nervous system require exploration. We investigated whether MEVs activate the heat shock response (HSR) in polarized human microglia. MEVs were isolated from unpasteurized human donor milk (n=12 anonymous donors). Human microglia clone 3 cells were primed with 10 ng/mL interferon-gamma to induce polarization, and a subset of cells was supplemented with 200 µg of MEVs. The abundance of HSF1 and candidate heat shock proteins (Hsp70, Hsp90, Hsp40, Hsp27) was analyzed using quantitative reverse transcription polymerase chain reaction and western immunoblotting at 6 h, 12 h, and 24 h post-MEV treatment. We found that MEV treatment promoted the HSR in polarized microglia, compared to homeostatic cells. Furthermore, MEVs increased the duration of the HSR in polarized microglia, exerting robust and continued pro-survival benefits.

## Introduction

Exposure to proteotoxic stress during perinatal (prenatal and postnatal) life can influence development and disease outcomes in offspring.^1^, ^2^, ^3^, ^4^ One of the principal outcomes of perinatal stress is neuroinflammation stemming from microglia polarization and the attenuation of cytoprotective, pro-survival cellular cascades.^5^, ^6^, ^7^ Microglia are the primary immune cells of the brain parenchyma and contribute to the innate and adaptive immune responses of the central nervous system (CNS).^7^, ^8^ Microglia are sensitive to stress and stimuli. Polarized microglia may respond to stimuli by secreting pro-inflammatory and anti-inflammatory factors in the CNS, which often result in changes in antigen presentation, cell proliferation, and/or morphology.^9^, ^10^

Recent studies indicate that human milk promotes offspring development and provides pro-survival benefits through the transmission of bioactive, regulatory molecules during critical periods of early life.^11^, ^12^, ^13^, ^14^, ^15^, ^16^, ^17^, ^18^ Milk-derived extracellular vesicles (MEVs) are immunomodulatory and anti-inflammatory^19^ with the potential to mitigate microglia functions and responses.^20^ MEVs are biological nanovesicles, ranging from 30 to 150 nm in diameter, that are secreted by cells of the mammary glands.^21^, ^22^ MEVs have a lipid bilayer which protects the encapsulated cargo against gastrointestinal degradation and low pH environments.^23^, ^24^ MEVs also travel *via* endocytosis and transcytosis across complex biological barriers, including the intestinal endothelium and the blood brain barrier.^11^, ^12^, ^23^, ^24^, ^25^, ^26^ The primary function of MEVs is to deliver their biologically active cargo, including deoxyribonucleic acid (DNA), messenger ribonucleic acid (mRNA), lipids, proteins, peptides, and small non-coding RNAs, such as microRNA (miRNA), to cells and tissues of the recipient.^21^, ^24^, ^27^

MEVs modulate developmental pathways, including epigenetic cascades, neurodevelopment, neurogenesis, and cell proliferation.^11^, ^27^, ^28^, ^29^, ^30^ MEVs also decrease rates of apoptosis in vitro and in vivo^31^, ^32^ and exhibit cytoprotective and anti-inflammatory effects in peripheral tissues, including the intestinal epithelium, hepatocytes, and the lungs.^33^, ^34^, ^35^, ^36^, ^37^ Specifically, MEVs have been shown to attenuate nuclear factor kappa B (NFκB) mediated inflammation, and prevent the progression of inflammatory diseases, such as necrotizing enterocolitis^33^, ^34^, ^35^, ^37^, ^38^, ^39^ and ulcerative colitis.^36^, ^40^ However, the cytoprotective potential of MEVs in human brain macrophages in response to neuroinflammation remains largely unknown. Specifically, the interaction between MEVs and existing pro-survival cascades needs further investigation to improve our understanding of how MEVs interact with existing mechanisms to enhance their cytoprotective function.

The heat shock response (HSR) is a foundational pro-survival mechanism that protects cells from environmental or pathophysiological challenges.^41^, ^42^ The HSR is regulated by DNA-binding heat shock-inducible transcription factors (HSFs), which regulate the transcription of molecular chaperones, called heat shock proteins (HSPs).^43^ HSPs reduce the aggregation of misfolded proteins, promote protein refolding, and direct misfolded proteins to the proteosome for proteolytic degradation.^43^ HSF1 is the master regulator of the HSR in human and murine systems.^42^, ^44^, ^45^, ^46^ In homeostatic cells, HSF1 transcription factor is constitutively found in the cytoplasm in the inactive monomeric state, where it associates with Hsp70 and Hsp90.^47^, ^48^ During proteotoxic stress, HSF1 dissociates from Hsp70 and Hsp90 and trimerizes.^49^, ^50^ Following trimerization, HSF1 becomes hyperphosphorylated, translocates to the nucleus, and binds to the 5′-nGAAn-3′ sequence (also referred to as the AGAAN heat shock element) of target genes.^46^, ^47^, ^51^, ^52^ HSF1 promotes the transcription of chaperones, including *HSPA* (i.e*.*, Hsp70), *HSPC* (i.e*.*, Hsp90), *DNAJ* (i.e*.*, Hsp40), and *HSPB* (small HSPs, e.g., Hsp27).^41^, ^43^

Hsp70 and Hsp90 are highly conserved adenosine triphosphate (ATP)-dependent molecular chaperones that together with HSP organizing protein (Hop) form the Hsp70–Hop–Hsp90 refolding complex.^48^, ^53^, ^54^, ^55^, ^56^ In addition to their role in restoring proteostasis, the Hsp70–Hop–Hsp90 complex represses HSF1 activation *via* negative feedback inhibition.^57^, ^58^ Hsp40 is a co-chaperone that transfers misfolded proteins to Hsp70 and stimulates Hsp70 ATPase activity.^59^, ^60^, ^61^ Hsp27 is involved in the rapid disaggregation of misfolded proteins, preventing them from forming aggregates until ATP-dependent chaperones are available.^45^, ^62^, ^63^, ^64^

The objective of this study is to further our understanding of the cytoprotective potential of MEVs, specifically in brain macrophages in response to an acute stress. We investigated the association between MEVs and the HSR in control and polarized human microglia. To our knowledge, this is the first study to explore the relationship between MEVs and baseline pro-survival pathways in the CNS, which is key to delineating the documented ability of human milk to reduce neuroinflammation and modulate neurodevelopment of infants.^65^, ^66^, ^67^, ^68^, ^69^

## Results

### The characterization and validation of MEV morphology, size, and biomarker profiles

**Fig. 1:**
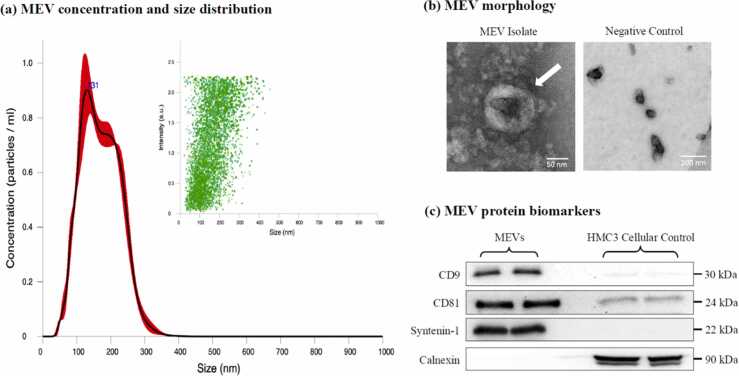
MEV characterization. (a) MEV concentration and size distribution were determined by nanoparticle tracking analysis (Malvern Instruments Ltd; NanoSight NS300). MEVs were diluted 1:300 (v/v in PBS) and readings were recording using an sCMOS camera (level 15, detection threshold 10) using a 532 nm green laser with 3× replicates at 30 s capture speeds. (b) MEV morphology was determined by transmission electron microscopy (Thermo Fisher Scientific; FEI Talos F200x S/TEM). MEVs were diluted 1:2 (v/v in PBS), loaded onto 400 mesh carbon-coated formvar film copper grids, and negatively stained using 2% uranyl acetate. (c) MEV protein biomarkers were detected using western immunoblotting. The endosome-specific transmembrane proteins CD9 and CD81, and the endosome-specific cytosolic protein syntenin-1 were used as positive controls. Calnexin, a marker of the endoplasmic reticulum, was used as a negative control to identify cellular protein contamination in the MEV isolates. Human microglia clone 3 (HMC3) represent the cellular control. Abbreviations used: MEV, milk-derived extracellular vesicle; PBS, phosphate-buffered saline.

We used nanoparticle tracking analysis (NTA) to measure MEV particle size and concentration. The isolated MEVs had a concentration of 2.13 × 10^11^ particles/mL with a mean size of 160 ± 0.5 nm (Figure 1(a)). MEV morphology was confirmed using transmission electron microscopy (TEM), allowing for distinct visualization of the electron-dense cargo and the lipid bilayer (Figure 1(b)). Some protein aggregates were detected alongside MEVs. No MEVs were detected in the negative control, but cellular debris was detected. NTA and TEM findings confirm that the isolated MEVs are within 30–150 nm.

Western immunoblotting was used to detect the presence of two tetraspanin proteins (CD9, CD81) and a protein involved in extracellular vesicle (EV) biogenesis (syntenin-1). CD9, CD81, and syntenin-1 were highly abundant in the MEV lysates, with reduced levels in the human microglia clone 3 (HMC3) cells (Figure 1(c)). Calnexin, an endoplasmic reticulum marker that was used as the negative control to represent cellular protein distribution, was absent from the MEV lysate and abundant in HMC3 lysate. The morphology and presence of positive endosome markers CD9, CD81, and syntenin-1, and distinct lack of calnexin, serve as a biological confirmation of our isolation and the lack of cellular contamination. HMC3 cells were used as the cellular control for MEV characterization.

### Cell viability remains unchanged in response to interferon-gamma and MEV treatment

**Fig. 2:**
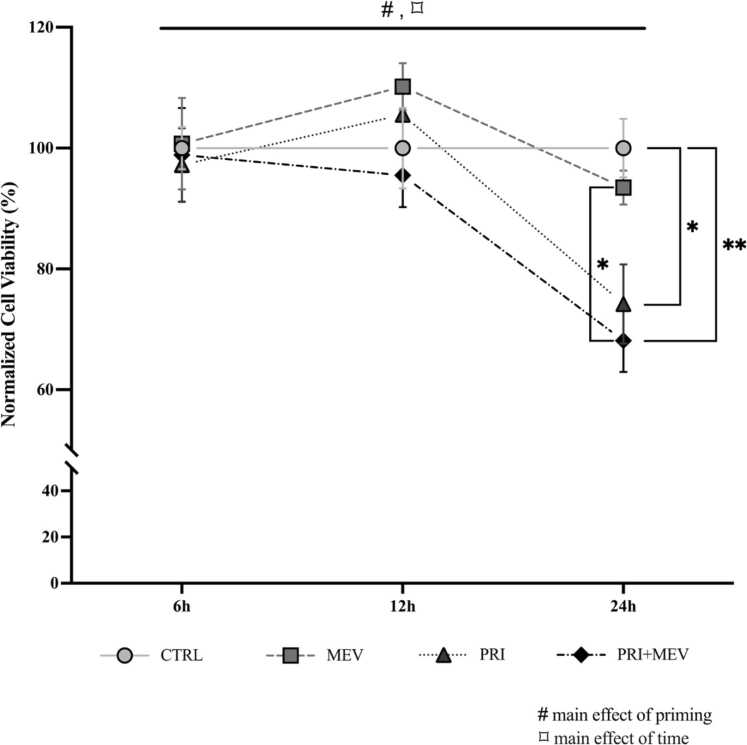
Cell viability with IFN-γ priming and MEV treatment in human microglia clone 3 cells. Cells were plated at 2.5 × 10^4^ cells/well. At >90% confluence, a subset of cells was treated with 10 ng/mL IFN-γ (PRI and PRI + MEV groups). Exactly 24 h post-IFN-γ treatment, a second subset of cells was supplemented with 200 µg MEV (MEV and PRI + MEV groups). A commercially available 3-(4, 5-dimethylthiazolyl-2)-2, 5-diphenyltetrazolium bromide assay was used to determine percent mitochondrial output and cell viability. The main effects of priming (denoted by ‘#’) and the main effects of time (denoted by ‘⌑’) are indicated. Significant differences between treatment groups were determined using a univariate general linear model with Tukey HSD (*P ≤ 0.05; **P < 0.01; ***P < 0.001). Abbreviations used: IFN-γ, interferon-gamma; MEV, milk-derived extracellular vesicle.

To determine if priming *via* the pro-inflammatory cytokine interferon-gamma (IFN-γ) and/or MEV treatment affected the mitochondrial oxidative output of HMC3 cells, we conducted an 3-[4,5-dimethylthiazol-2-yl]−2,5 diphenyl tetrazolium bromide (MTT) assay at 6, 12, and 24 h (Figure 2). At 6 h, cell viability remained unchanged across treatment groups (main effect of priming: (*F*_(__1__,__16__)_ = 0.126, *P* = 0.727); main effect of MEV treatment: (*F*_(__3__,__16__)_ = 0.057, *P* = 0.981)). Specifically, the cell viability (mean ± standard error of mean) at 6 h was 100.00% ± 3.50% for control (CTRL), 100.73% ± 7.58% for MEV, 97.21% ± 6.08% for primed (PRI), and 98.86% ± 7.76% for PRI + MEV. At 12 h, cell viability also remained unchanged across treatment groups (main effect of priming: (*F*_(__1__,__15__)_ = 0.241, *P* = 0.630); main effect of MEV treatment: (*F*_(__3__,__15__)_ = 1.027, *P* = 0.409)). The cell viability at 12 h was 100.00% ± 6.68% for CTRL, 110.16% ± 3.89% for MEV, 105.53% ± 4.48% for PRI, and 95.48% ± 5.27% for PRI + MEV. At 24 h, there was a main effect of priming (*F*_(__1__,__14__)_ = 22.032, *P* < 0.001) where PRI cells (Tukey HSD, *P* = 0.015) and PRI + MEV (Tukey HSD, *P* = 0.005) cells had a lower cell viability compared to CTRL cells, and where PRI + MEV cells had a lower cell viability compared to MEV cells (Tukey HSD, *P* = 0.034). There is a main effect of time (*F*_(__2,46__)_ = 11.644, *P* < 0.001), where cells at 24 h had a lower cell viability compared to 6 h cells (Tukey HSD, *P* = 0.002) and 12 h cells (Tukey HSD, *P* < 0.001). The cell viability at 24 h was 100.00% ± 4.85% for CTRL, 93.46% ± 2.81% for MEV, 74.20% ± 6.54% for PRI, and 68.12% ± 5.18% for PRI + MEV.

### MEV treatment increases HSF1 protein abundance

**Fig. 3:**
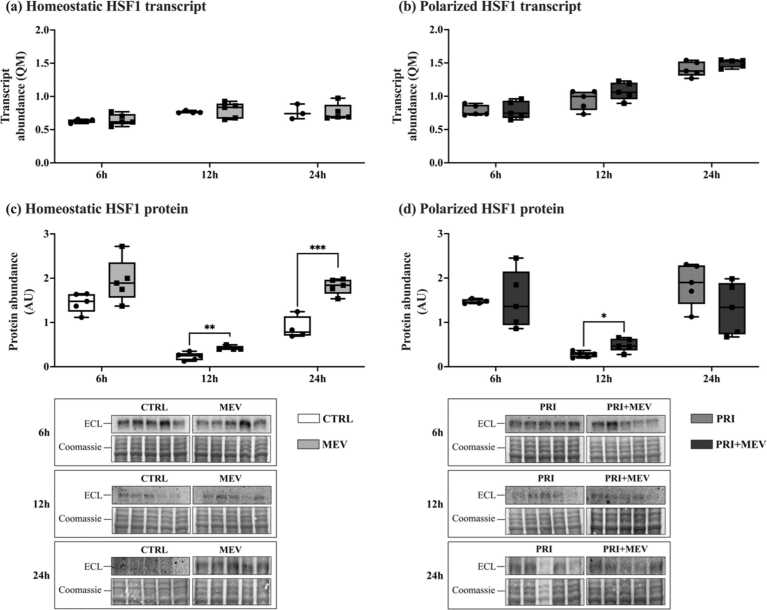
Transcript and protein abundance of HSF1 in response to IFN-γ priming and MEV treatment, in homeostatic (CTRL and MEV) and polarized (PRI and PRI + MEV) human microglia clone 3 cells. (a) Homeostatic HSF1 transcript abundance. (b) Polarized HSF1 transcript abundance. (c) Homeostatic HSF1 protein abundance. (d) Polarized HSF1 protein abundance. RT-qPCR targets are normalized to the geometric mean of reference genes: *GAPDH* and *PKM*. Protein targets are normalized to total protein levels, with ECL and Coomassie-stained blot images displayed. Significant differences between treatment groups were determined using a two-tailed, independent samples t-test with Tukey HSD (*P ≤ 0.05; **P < 0.01; ***P < 0.001). Abbreviations used: ECL, enhanced chemiluminescence; IFN-γ, interferon-gamma; MEV, milk-derived extracellular vesicle; QM, quantity mean; RT-qPCR, quantitative reverse transcription polymerase chain reaction.

Transcript abundance of HSF1 remained unchanged in homeostatic microglia at 6 h (*t*(7) = −0.429, *P* = 0.681), 12 h (*t*(7) = −4.10, *P* = 0.666), and 24 h (*t*(5) = 0.927, *P* = 0.396) with MEV treatment (Figure 3(a)). Similarly, in polarized microglia, no changes were seen in HSF1 transcript at 6 h (*t*(8) = −0.080, *P* = 0.938), 12 h (*t*(8) = −1.478, *P* = 0.178), and 24 h (*t*(8) = −1.498, *P* = 0.173) (Figure 3(b)).

At the protein level, in homeostatic microglia, HSF1 remained unchanged at 6 h (*t*(8) = −2.055, *P* = 0.074), but increased with MEV treatment at 12 h (*t*(7) = −3.811, *P* = 0.007) and 24 h (*t*(7) = −6.549, *P* < 0.001) (Figure 3(c)). Similarly, in polarized microglia, HSF1 abundance increased with MEV treatment at 12 h (*t*(8) = −2.991, *P* = 0.017), but remained unchanged at 6 h (*t*(7) = −0.111, *P* = 0.906) and 24 h (*t*(8) = 1.610, *P* = 0.146) (Figure 3(d)).

**Fig. 4:**
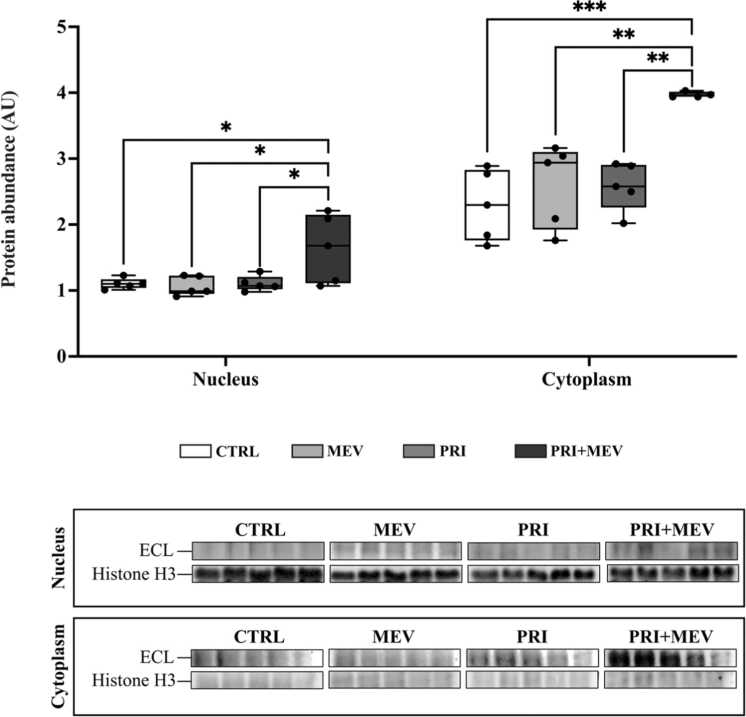
HSF1 protein localization from the cytoplasm to the nucleus in response to IFN-γ priming and MEV treatment in homeostatic (CTRL and MEV) and polarized (PRI and PRI + MEV) human microglia clone 3 cells. Protein targets are normalized to total protein levels, with ECL and Coomassie-stained blot images displayed. Significant differences between treatment groups were determined using a one-way ANOVA with Tukey HSD (*P ≤ 0.05; **P < 0.01; ***P < 0.001). Abbreviations used: ANOVA, one-way analysis of variance; ECL, enhanced chemiluminescence; IFN-γ, interferon-gamma; MEV, milk-derived extracellular vesicles.

To examine HSF1 nuclear translocation (indicative of enhanced transcriptional activity), we assessed total HSF1 levels in the cytoplasm and the nucleus of HMC3 cells at 12 h post-MEV treatment. 12 h post-supplementation was chosen for this analysis because the most robust changes in HSF1 protein levels were seen at the 12 h timepoint in homeostatic and polarized microglia. In PRI + MEV cells, HSF1 was highly abundant in the nucleus (*F*_(__3__,__16__)_ = 4.787, *P* = 0.014) compared to CTRL (Tukey HSD, *P* = 0.037), MEV (Tukey HSD, *P* = 0.025), and PRI (Tukey HSD, *P* = 0.037) cells (Figure 4). An increased abundance of HSF1 was also detected in the cytoplasm in PRI + MEV cells (*F*_(__3__,__15__)_ = 10.929, *P* < 0.001) compared to CTRL (Tukey HSD, *P* < 0.001), MEV (Tukey HSD, *P* = 0.003) and PRI (Tukey HSD, *P* = 0.003) cells (Figure 4). The presence of histone H3, an integral component of the nucleosome, in the nuclear fraction and its absence in the cytoplasmic fraction confirms proper isolation of the cellular compartments.

### 2.4 MEV treatment decreases Hsp70 protein abundance

**Fig. 5:**
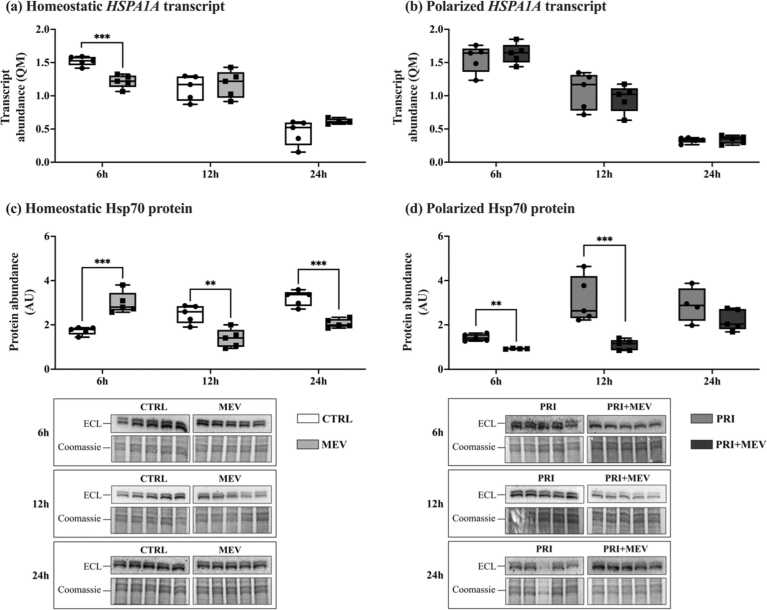
Transcript and protein abundance of Hsp70 (*HSPA1A*) in response to IFN-γ priming and MEV treatment, in homeostatic (CTRL and MEV) and polarized (PRI and PRI + MEV) human microglia clone 3 cells. (a) Homeostatic *HSPA1A* transcript abundance. (b) Polarized *HSPA1A* transcript abundance. (c) Homeostatic Hsp70 protein abundance. (d) Polarized Hsp70 protein abundance. RT-qPCR targets are normalized to the geometric mean of reference genes: *GAPDH* and *PKM*. Protein targets are normalized to total protein levels, with ECL and Coomassie-stained blot images displayed. Significant differences between treatment groups were determined using a two-tailed, independent samples t-test with Tukey HSD (*P ≤ 0.05; **P < 0.01; ***P < 0.001). Abbreviations used: ECL, enhanced chemiluminescence; IFN-γ, interferon-gamma; MEV, milk-derived extracellular vesicle; QM, quantity mean; RT-qPCR, quantitative reverse transcription polymerase chain reaction.

In homeostatic cells, the transcript abundance of *HSPA1A* decreased with MEV treatment at 6 h (*t*(8) = 5.599, *P* < 0.001), but remained unchanged at 12 h (*t*(8) = −0.433, *P* = 0.677) and 24 h (*t*(7) = −1.726, *P* = 0.118) (Figure 5(a)). In polarized cells, there were no changes in transcript abundance of *HSPA1A* across the 6 h (*t*(8) = −0.694, *P* = 0.507), 12 h (*t*(8) = 0.726, *P* = 0.489), and 24 h (*t*(8) = −0.217, *P* = 0.834) timepoints (Figure 5(b)).

At the protein level, in homeostatic cells, Hsp70 levels increased at 6 h (*t*(8) = −5.466, *P* < 0.001), but decreased at 12 h (*t*(8) = 4.177, *P* = 0.003) and 24 h (*t*(8) = 6.331, *P* < 0.001) (Figure 5(c)). In polarized cells, Hsp70 decreased at 6 h (*t*(7) = 6.462, *P* = 0.002) and 12 h (*t*(8) = 4.267, *P* = 0.010), but no change was observed at 24 h (*t*(7) = 1.642, *P* = 0.145) (Figure 5(d)).

### MEV treatment decreases Hsp90 protein abundance

**Fig. 6:**
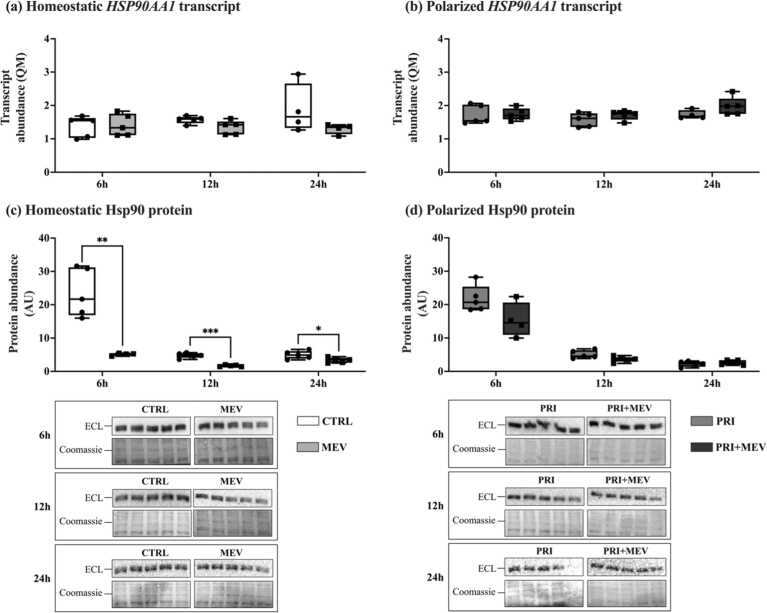
Transcript and protein abundance of Hsp90 (*HSP90AA1*) in response to IFN-γ priming and MEV treatment, in homeostatic (CTRL and MEV) and polarized (PRI and PRI + MEV) human microglia clone 3 cells. (a) Homeostatic *HSP90AA1* transcript abundance. (b) Polarized *HSP90AA1* transcript abundance. (c) Homeostatic Hsp90 protein abundance. (d) Polarized Hsp90 protein abundance. RT-qPCR targets are normalized to the geometric mean of reference genes: *GAPDH* and *PKM*. Protein targets are normalized to total protein levels, with ECL and Coomassie-stained blot images displayed. Significant differences between treatment groups were determined using a two-tailed, independent samples t-test with Tukey HSD (*P ≤ 0.05; **P < 0.01; ***P < 0.001). Abbreviations used: ECL, enhanced chemiluminescence; IFN-γ, interferon-gamma; MEV, milk-derived extracellular vesicle; QM, quantity mean; RT-qPCR, quantitative reverse transcription polymerase chain reaction.

MEV treatment did not change transcript abundance of *HSP90AA1* in homeostatic cells at 6 h (*t*(8) = −0.211, *P* = 0.838), 12 h (*t*(8) = 2.132, *P* = 0.066), and 24 h (*t*(7) = 1.677, *P* = 0.128) (Figure 6(a)). Similarly, transcript abundance did not change in polarized cells at 6 h (*t*(8) = −0.119, *P* = 0.908), 12 h (*t*(8) = −1.225, *P* = 0.255), and 24 h (*t*(7) = −1.695, *P* = 0.134) (Figure 6(b)).

At the protein level, MEV treatment led to decreases in Hsp90 in homeostatic cells at the 6 h (*t*(7) = 4.988, *P* = 0.005), 12 h (*t*(8) = 8.526, *P* < 0.001), and 24 h (*t*(8) = 2.469, *P* = 0.039) timepoints (Figure 6(c)). No changes were observed in polarized cells at 6 h (*t*(7) = 2.091, *P* = 0.075), 12 h (*t*(8) = 2.270, *P* = 0.053), or 24 h (*t*(8) = −0.860, *P* = 0.415) (Figure 6(d)).

### MEV treatment increases Hsp40 protein abundance

**Fig. 7:**
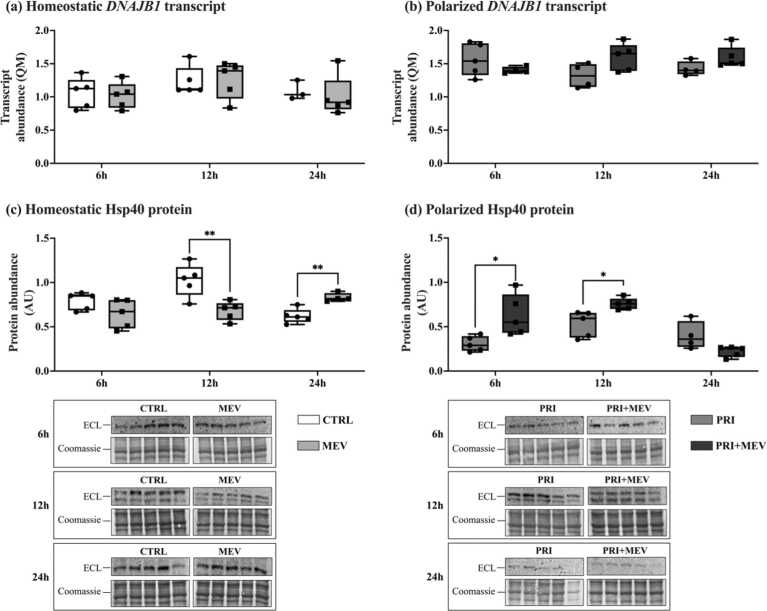
Transcript and protein abundance of Hsp40 (*DNAJB1*) in response to IFN-γ priming and MEV treatment, in homeostatic (CTRL and MEV) and polarized (PRI and PRI + MEV) human microglia clone 3 cells. (a) Homeostatic *DNAJB1* transcript abundance. (b) Polarized *DNAJB1* transcript abundance. (c) Homeostatic Hsp40 protein abundance. (d) Polarized Hsp40 protein abundance. RT-qPCR targets are normalized to the geometric mean of reference genes: *GAPDH* and *PKM*. Protein targets are normalized to total protein levels, with ECL and Coomassie-stained blot images displayed. Significant differences between treatment groups were determined using a two-tailed, independent samples t-test with Tukey HSD (*P ≤ 0.05; **P < 0.01; ***P < 0.001). Abbreviations used: ECL, enhanced chemiluminescence; IFN-γ, interferon-gamma; MEV, milk-derived extracellular vesicle; QM, quantity mean; RT-qPCR, quantitative reverse transcription polymerase chain reaction.

Transcript abundance of *DNAJB1* remained unchanged with MEV treatment in homeostatic cells at 6 h (*t*(8) = 0.304, *P* = 0.769), 12 h (*t*(8) = −0.134, *P* = 0.897), and 24 h (*t*(5) = 2.548, *P* = 0.051) (Figure 7(a)). Similarly, transcript abundance did not change in polarized cells at 6 h (*t*(7) = 1.258, *P* = 0.224), 12 h (*t*(8) = −0.918, *P* = 0.385), and 24 h (*t*(7) = −1.798, *P* = 0.115) (Figure 7(b)).

At the protein level, Hsp40 remained unchanged in homeostatic cells at 6 h (*t*(8) = 1.689, *P* = 0.130), but we observed a decrease in abundance at 12 h (*t*(8) = 3.616, *P* = 0.007), followed by an increase at 24 h (*t*(7) = −4.497, *P* = 0.003) (Figure 7(c)). In polarized cells, Hsp40 increased at both 6 h (*t*(8) = −2.894, *P* = 0.034) and 12 h (*t*(8) = −3.188, *P* = 0.021), but remained unchanged at 24 h (*t*(8) = 0.958, *P* = 0.366) (Figure 7(d)).

### MEV treatment leads to differential Hsp27 protein abundance

**Fig. 8:**
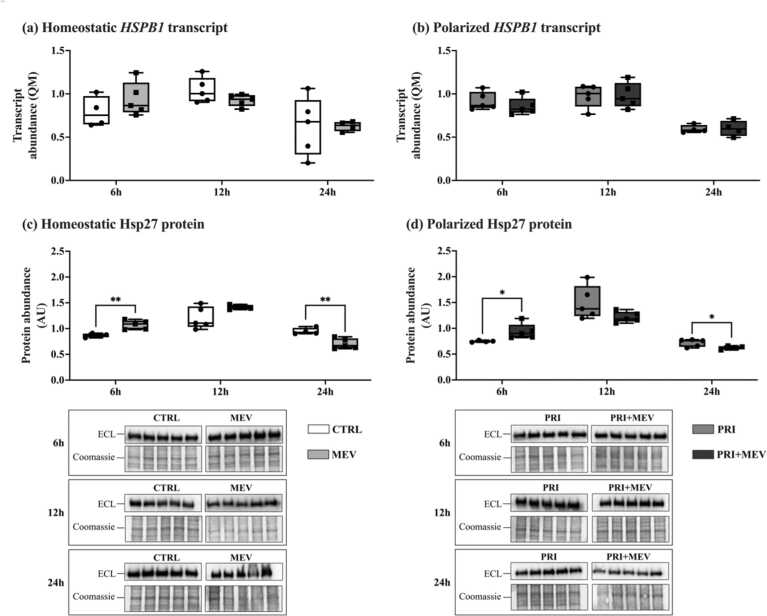
Transcript and protein abundance of Hsp27 (*HSPB1*) in response to IFN-γ priming and MEV treatment, in homeostatic (CTRL and MEV) and polarized (PRI and PRI + MEV) human microglia clone 3 cells. (a) Homeostatic *HSPB1* transcript abundance. (b) Polarized *HSPB1* transcript abundance. (c) Homeostatic Hsp27 protein abundance. (d) Polarized Hsp27 protein abundance. RT-qPCR targets are normalized to the geometric mean of reference genes: *GAPDH* and *PKM*. Protein targets are normalized to total protein levels, with ECL and Coomassie-stained blot images displayed. Significant differences between treatment groups were determined using a two-tailed, independent samples t-test with Tukey HSD (*P ≤ 0.05; **P < 0.01; ***P < 0.001). Abbreviations used: ECL, enhanced chemiluminescence; IFN-γ, interferon-gamma; MEV, milk-derived extracellular vesicle; QM, quantity mean; RT-qPCR, quantitative reverse transcription polymerase chain reaction.

Transcript abundance of *HSPB1* remained unchanged with MEV treatment in homeostatic cells at 6 h (*t*(7) = −1.179, *P* = 0.277), 12 h (*t*(8) = 1.591, *P* = 0.150), and 24 h (*t*(7) = −0.002, *P* = 0.998) (Figure 8(a)). No changes were observed in polarized cells at 6 h (*t*(7) = 0.959, *P* = 0.365), 12 h (*t*(8) = −0.072, *P* = 0.945), or 24 h (*t*(6) = −0.119, *P* = 0.909) as well (Figure 8(b)).

Hsp27 protein abundance increased in homeostatic cells at 6 h (*t*(7) = −4.293, *P* = 0.004), however, no change was observed at 12 h (*t*(7) = −1.916, *P* = 0.091), and interestingly Hsp27 protein levels decreased at 24 h (*t*(7) = 4.166, *P* = 0.004) (Figure 8(c)). In polarized cells, the same pattern was observed where Hsp27 protein levels increased with MEV treatment at 6 h (*t*(7) = −2.484, *P* = 0.042), with no change at 12 h (*t*(8) = 1.853, *P* = 0.125), and decreased at 24 h (*t*(7) = 2.270, *P* = 0.050) (Figure 8(d)).

## Discussion

MEVs have immunomodulatory and anti-inflammatory properties. While MEVs have been shown to attenuate pro-inflammatory pathways and reverse proteotoxicity in peripheral tissues and organs, their bioactivity in immune-reactive brain macrophages remains to be investigated. We investigated if and how MEVs may interact with the basal HSR, a principal pro-survival response, in homeostatic and IFN-γ polarized human microglia. Our findings suggest that MEV treatment promotes the HSR by increasing HSF1 abundance and prolonging its activity.

To ensure that IFN-γ priming and MEV treatment did not impact cell viability and metabolic output, we conducted an MTT assay. The cell viability remained unchanged at 6 h and 12 h in cells that received both treatments compared to control cells, suggesting that IFN-γ priming and MEV treatment do not change the overall metabolic activity of the HMC3 cells (Figure 2). At 12 h, MEV treatment leads to a slight increase in cell viability (above 100%), compared to the control untreated cells. Live cells with an active metabolism use mitochondrial dehydrogenases to reduce the MTT reagent into formazan, which can be analyzed as a proxy of cell viability.^70^ Since formazan is insoluble and can accumulate as crystals inside the cells,^70^ likely the increase in absorbance with MEV treatment that we are seeing at 12 h is due to formazan crystal aggregation rather than a biologically relevant increase. As such, this increase should not be interpreted as an increase in cellular proliferation, viability, or mitochondrial output. Our results highlight key limitations of MTT-based cell viability assays and the necessity for optimization and rational interpretation of results.^71^ At 24 h, a significant priming effect is evident, where the PRI and PRI + MEV groups have reduced cell viability compared to the untreated homeostatic cells and homeostatic cells receiving MEVs. . Contrary to our findings, a study by La Torre *et al.*^72^ reported that administration of EVs (10 μg/mL) isolated from immortalized mouse microglia (BV-2) for 24 h did not impact the cell viability of control BV-2 cells, or BV-2 cells pre-treated for 24 h with 1 µg/mL LPS. Tong *et al.*^36^ also reported that 24 h incubation with isolated bovine MEVs (30–480 μg/mL) and LPS (100 ng/mL) did not affect the cell viability of RAW264.7 macrophage-like cells. It is possible that the reason we see a decrease in cell viability at 24 h is due to the unique responses of immortalized human microglia to stress, which can depend on the cell type, culture age, developmental stage, and host organism, as well as the type of stimulus, dosage, and duration.^73^

HSF1 is the primary transcription factor that regulates the expression of HSP chaperones in response to and in preparation for proteotoxic stress.^74^ HSF1 is typically expressed very early during cellular stress and is cell-specific and time-specific. Thus, HSF1 transcriptional output can be difficult to capture.^75^ Correspondingly, at the transcript level, no significant changes are observed in homeostatic and polarized microglia at 6 h, 12 h, and 24 h post-MEV treatment (Figure 3(a) and (b)). Given that an immediate transcriptional response is required upon initiation of the HSR, HSF1 activation is primarily regulated at the protein level.^50^ As a result, HSF1 protein tends to change more drastically than mRNA, especially in experimental conditions with low temporal resolution, potentially resulting in discrepancies between mRNA and protein abundance data.^75^ This is largely due to dynamic post-transcriptional (miRNA) and post-translational regulatory mechanisms that shape gene expression patterns. The fact that the earliest timepoint we analyzed in our study was 6 h post-MEV treatment, it is likely that we had missed the transcriptional regulation but were able to capture changes in protein regulation. At the protein level, HSF1 increases in abundance in homeostatic microglia at 12 h and 24 h and increases in polarized microglia at 12 h (Figure 3(c) and (d)).

Cytonuclear translocation of HSF1 shows that HSF1 abundance in the cytoplasmic and nuclear compartments is highest in polarized cells receiving MEVs (Figure 4), compared to homeostatic cells. This suggests that HSF1 is not only increasing in polarized microglia, but that MEV treatment in polarized cells leads to higher levels of active HSF1 in the nucleus. Taken together, our data suggest that MEV treatment may increase HSR activation and enhance cytoprotective, pro-survival responses in polarized HMC3 cells. Liao *et al.*^76^ report that in a murine model of Parkinson’s disease, overexpression of HSF1 represses microglia polarization, increases the abundance of anti-inflammatory markers (transforming growth factor 1 beta and interleukin 10), and decreases the abundance of pro-inflammatory markers (interleukin 1 beta, interleukin 6, and tumor necrosis factor alpha (TNFα)), when compared to Parkinson’s disease-induced microglia. Furthermore, Janus *et al.*^77^ report that HSF1 can attenuate pro-inflammation by inhibiting candidate genes involved in the NFκB pathway, including NFκB inhibitor delta, TNF receptor-associated factor 2, and TNF receptor-associated factor 3 in human MCF7 ERα-positive breast cancer cells.

Similar to HSF1, the induction of HSP chaperones is primarily regulated at the protein level.^50^ The Hsp70 chaperone conducts protein folding and disaggregation and is an integral component of the Hsp70–Hop–Hsp90 complex.^78^, ^79^ We observe a decrease in *HSPA1A* transcript levels in homeostatic microglia at 6 h (Figure 5(a)), while *HSPA1A* transcript levels remained unchanged across other time points and in polarized microglia (Figure 5(a) and (b)). A potential explanation for this decrease in *HSPA1A* transcript at 6 h is that MEV miRNA may induce post-transcriptional regulation of Hsp70 post-MEV treatment. Several studies suggest that miRNAs are the most likely candidates within MEVs to induce post-transcriptional regulation in recipient cells and systems.^80^, ^81^, ^82^ However, the MEV miRNA-induced post-transcriptional regulation of HSP chaperones in the brain requires further study.

The decrease in *HSPA1A* transcript at 6 h in the homeostatic microglia is consistent with the Hsp70 protein levels at 12 h and 24 h (Figure 5(c)). Baseline homeostatic responses are typically activated earlier at the gene level, and these changes subsequently appear at the protein level several minutes to hours later. Further, as an integral part of the Hsp70–Hop–Hsp90 complex, Hsp70 plays a role in the negative regulation of HSF1 activation.^50^ Hsp70 is responsible for interfering with the binding and stabilization of HSF1 monomers, preventing trimerization, and thus activation of HSF1.^50^ This negative feedback loop is designed to limit the hyperactivation and prolonged function of HSF1. At 12 h and 24 h, while Hsp70 protein levels decrease, HSF1 protein abundance increases in the same cells (Figure 3(a)). Likely, Hsp70 downregulation is resulting in the upregulation of HSF1. However, we observed a peculiar trend with Hsp70 at the protein level at 6 h in homeostatic cells, where Hsp70 protein abundance increases, unlike the decrease observed at 12 h and 24 h (Figure 5(c)). Hsp70 is highly enriched in MEVs.^83^, ^84^, ^85^ As such, the increase we observe is likely a combinatorial effect of MEV containing Hsp70 treatment of the HMC3 cells that contain endogenously produced Hsp70, rather than an increase in Hsp70 protein translation in HMC3 cells in response to the treatments.

In polarized cells, we see a decrease in Hsp70 abundance at 6 h and 12 h, like the homeostatic 12 h and 24 h cells (Figure 5(d)). Once again, the decrease in Hsp70 abundance in polarized cells complements the increase in HSF1. This suggests that MEVs may be potentiating and prolonging HSF1 activity by reducing the amount of Hsp70 available in the cytoplasm. Interestingly, although not statistically tested, we observed an inverse relationship between HSPA*1A* transcript levels that decreases with time (from 6 h to 24 h) (Figure 5(b)), while the transcript abundance of HSF1 increases with time in polarized microglia (Figure 3(b)).

The Hsp90 chaperone is another key regulator of proteostasis and is a member of the Hsp70–Hop–Hsp90 complex.^86^ As a binding partner within the Hsp70–Hop–Hsp90 complex, Hsp90 is involved in the disassembly of HSF1 trimers and attenuates HSF1-mediated gene expression of candidate HSPs.^50^ Our results show that with MEV treatment, a similar decreasing pattern observed in Hsp70 is also observed in Hsp90 (Figure 6(a) and (b)), with a corresponding increase in HSF1. These results suggest that MEV treatment may lead to the downregulation of Hsp70 and Hsp90 to promote the prolonged activation of HSF1.^87^, ^88^, ^89^, ^90^, ^91^

Hsp40 is a co-chaperone that identifies and delivers misfolded proteins to Hsp70 for refolding by the Hsp70–Hop–Hsp90 complex.^92^ We did not observe significant differences in Hsp40 transcript levels in baseline or polarized microglia across timepoints (Figure 7(a) and (b)). At the protein level, in homeostatic cells, Hsp40 abundance decreases at 12 h (Figure 7(c)). This follows the same pattern as homeostatic Hsp70 abundance at 12 h. Generally, Hsp40 and Hsp70 follow similar regulatory patterns because Hsp40 enhances the ATPase activity of Hsp70.^93^, ^94^ However, we did not see a positive association between Hsp70 and Hsp40 protein levels at 24 h in homeostatic cells, nor at 6 h and 12 h in polarized cells. At these timepoints, Hsp40 protein abundance increases with MEV treatment (Figure 7(c) and (d)). Although the reason underlying this inverse regulation of Hsp70 and Hsp40 remains to be characterized, our study is not the first to report an inverse correlation. Chand *et al.*^95^ report that during HIV-1 infection, Hsp70 (*HSPA1A*) was downregulated, whereas Hsp40 (*DNAJB1*) was upregulated. This suggests that the association between Hsp70 and Hsp40 may be cell-specific, tissue-specific, and stress-specific. As a possible explanation, we postulate that instead of increasing the ATPase activity of Hsp70, Hsp40 may be interacting with heat shock cognate 70 (Hsc70).^96^, ^97^ Hsc70 is also a part of the *HSPA* family (encoded by *HSPA8*), although it is constitutively expressed and is localized primarily in the cytoplasm.^98^ Hsc70 is the only known protein involved in chaperone-mediated autophagy, where misfolded clients are brought to the lysosome for degradation.^99^ Hsp40 has been reported to stimulate Hsc70 ATPase activity, playing a critical role in Hsc70 substrate binding, and thus in preventing protein aggregation *via* chaperone-mediated autophagy.^97^ Further research is required to determine the specific Hsp40 binding partners in homeostatic and polarized microglia with MEV-supplementation.

Hsp27 is an ATP-independent chaperone that is integral in the rapid disaggregation of nuclear proteins.^45^, ^62^ No changes are observed in Hsp27 transcript level in homeostatic or polarized microglia (Figure 8(a) and (b)). At the protein level, Hsp27 increases in homeostatic and polarized cells at 6 h, with no changes at 12 h, but decreases at 24 h (Figure 8(c) and (d)). In the absence of ATP, Hsp27 acts as a “holdase” and helps solubilize misfolded proteins, remodeling them into intermediate forms to be refolded by Hsp70 or Hsp90, and minimizing protein aggregation.^63^, ^64^ Given the temporal importance in Hsp27 activation as an ATP-independent chaperone, we postulate that its upregulation during the early stages of proteotoxic stress is critical; however, once other ATP-dependent chaperones are established, Hsp27-mediated protein rescuing becomes less crucial for maintaining homeostasis. Further studies are required to delineate the mechanisms of Hsp27 regulation with MEV treatment.

## Conclusion

The cytoprotective and anti-inflammatory properties of MEVs have been largely established in peripheral tissues and organs in vitro and in vivo. Our study is the first to investigate the interactions between MEVs and pro-survival pathways in HMC3 cells. We found that MEV treatment promotes the HSR in homeostatic and polarized human microglia. Specifically, MEV treatment increases the abundance of HSF1, the main transcription factor, while downregulating the formation of Hsp70–Hop–Hsp90 complex. Since Hsp70 and Hsp90 are implicated in the negative regulation of HSF1, their downregulation with MEV treatment allows for prolonged HSR activation, exerting robust and continued pro-survival benefits in response to IFN-γ-induced polarization in microglia. However, our study does not explore the mechanisms behind how MEV cargo may regulate the HSR. While additional components of MEV cargo may contribute to HSR regulation (e.g., short-chain fatty acids and peptides), it is likely that miRNAs play a significant regulatory role. Future studies should explore MEV miRNAs that interact with and induce the downregulation of *HSPC* (e.g., *HSP90AA1*) and *HSPA* family genes (e.g.*, HSPA1A*). Among the top five enriched MEV miRNAs to further test are hsa-miR-148a-3p, hsa-miR-30d-5p, hsa-miR-200a-3p, and hsa-miR-let-7b-5p.^100^ Specifically, *HSPA* genes have been previously reported to be downregulated specifically by hsa-miR-30d-5p,^88^, ^101^ and *HSPC* genes have been reported to be downregulated by hsa-miR-148a-3p, hsa-miR-200a-3p, and hsa-miR-let-7b-5p.^87^, ^88^, ^89^ Although these studies were not conducted in HMC3 cells, pathophysiological conditions, or in the context of MEV treatment, it is a promising avenue for future research.

## Materials and methods

### MEV isolation and characterization

Unpasteurized human donor milk (n = 11 anonymous donors) was obtained from NorthernStar Mothers Milk Bank (Calgary, Alberta, Canada) and pooled to create a homogeneous mixture. The milk was pooled to limit individual variability in milk composition across donors (e.g., metabolic/health status, milk stage). The milk was serially centrifuged to remove creams, fats, and milk fat globular membranes (twice at 3000× g, 10 mins, 22 °C), cellular debris (twice at 1,200× g, 10 mins, 4 °C), and milk cells (twice at 21,500× g, 30 mins, 4 °C, followed by once at 21,500× g, 60 mins, 4 °C). The remaining whey fraction was filtered using a 0.45 µm syringe filter (UltiDent Scientific Inc., Rue Locke, Quebec, CA: 229749) to remove residual milk cells and debris. Casein protein was removed using acetic acid precipitation (1:1000, v/v), and the whey supernatant was centrifuged (4500× g, 30 mins, 4 °C) and filtered through a 0.22 µm syringe filter (UltiDent: 229747) to remove casein proteins.^102^ To isolate the MEVs, the supernatant was ultracentrifuged at 100,000× g for 120 mins at 4 °C using the XL-100 ultracentrifuge with a SW55TI swing bucket rotor, no breaks (Beckman-Coulter, Brea, California, USA). The MEV pellet was washed in filtered 1X phosphate-buffered saline (PBS) (1:1, v/v) and a second ultracentrifugation was completed at the same settings. The MEV pellet was resuspended in filtered 1X PBS (1:2, v/v to original whey volume) to create an enriched MEV sample used for supplementation experiments, and the supernatant from the second ultracentrifugation step was used as an EV-depleted negative control.^22^

Following isolation, MEVs were characterized in accordance with the Minimum information for studies of extracellular vesicles 2023 guidelines.^103^ MEV particle size and concentration, and negative controls were quantified using the NanoSight NS300 (Malvern Instruments Ltd, Malvern, UK) at the Hospital for Sick Children’s Structural and Biophysical Core Facility (Toronto, Ontario, CA). MEVs were diluted (1:300, v/v in 1X filtered PBS)^31^ using an sCMOS camera (level 15, detection threshold 10) using a 532 nm green laser (3× replicates at 30 s capture speeds) as per Wijenayake *et al.*^22^

MEV morphology and integrity were characterized using the FEI Talos F200x S/TEM (Thermo Fisher Scientific, Waltham, Massachusetts, USA) as per Wijenayake *et al.*^22^ at the Manitoba Institute of Materials (Winnipeg, Manitoba, CA). MEVs were loaded onto 400 mesh carbon-coated formvar film copper grids (Electron Microscopy Sciences, Hatfield, Pennsylvania, USA: CF400-CU-50) suspended in 1X PBS (1:3, v/v), and negatively stained using 2% uranyl acetate.

The presence of three endosome-specific proteins, including two tetraspanins (CD9, CD81) and an EV biogenesis factor (syntenin-1), was detected using western immunoblotting. Protein abundance of calnexin, a cellular marker of the endoplasmic reticulum, was used as the negative control to identify cellular protein contamination in the MEV isolates. Total soluble protein isolated from HMC3 cells was used as a cellular control. Total soluble protein was extracted by incubating MEVs and HMC3 cells in cell extraction buffer (Thermo Fisher Scientific: FNN0011), protease inhibitor cocktail (1:20, v/v) (BioShop, Burlington, Ontratio, CA: PIC001.1) and phenylmethanesulfonyl fluoride (1 mM) (BioShop: PMS123.5) for 30 min on ice with intermittent vortexing, followed by centrifugation (13,000 rpm, 10 min, 4 °C). Protein concentrations were quantified *via* a bicinchoninic acid (BCA) assay using the Pierce™ BCA Protein Assay kit (Thermo Fisher Scientific: 23227), according to the manufacturer’s instructions. Absorbance readings were obtained at 562 nm using a BioTek Synergy H1 Multimode microplate reader (Agilent, Santa Clara, California, USA: BTSH1M2SI). Bromophenol blue loading dye containing sodium dodecylsulfate (SDS) (Bioshop: SDS001.1) and *β-*mercaptoethanol (10%, v/v) (Bioshop: MERC002.500) was added to the samples, vortexed, and heated to 95 °C for 10 min to denature the proteins. Converted lysates were stored at −20 °C. Western immunoblotting parameters for MEV protein biomarkers are listed in Table S1. Antibody information for MEV biomarkers is listed in Table S2. Enhanced chemiluminescence (ECL) and Coomassie images for MEV protein biomarkers can be found in Figure S1. Isolated MEVs were stored at −80 °C for supplementation experiments.

### HMC3 culture

Immortalized HMC3 cell line (American Type Culture Collection (ATCC), Manassas, Virginia, USA: CRL-3304; lot number: 70026037) was donated by Dr. Patrick O. McGowan (1 vial; Passage (P) 1; 2.26 × 10^6^ cells/mL) from the University of Toronto. The transfer of the culture to Dr. Sanoji Wijenayake at The University of Winnipeg (received on dry ice, February 14th, 2023) followed permit requirements and material transfer agreements from ATCC and the Canadian Food Inspection Agency (compliance letter ID: CL-2022-0019-4). Cells were cultured with Eagle’s Minimum Essential Media (ATCC: 30-2003) supplemented with 10% fetal bovine serum (FBS) (ATCC: 30-2020) and maintained in a humidified incubator at 37 °C with 5% CO_2_, as per the manufacturer’s recommendations. Complete media was replaced every 48 h to prevent excessive acidification.^104^ Exactly 0.25% Trypsin with 0.52 nM ethylendiaminetetraacetic acid (EDTA) (ATCC: 30-2101) was used for cell detachment and harvesting. A hemocytometer was used for manual cell counting. Specifically, live cells in each of the four quadrants were manually counted, and cell density and total cell counts were determined using the following formulae:

(1) $$\text{Cell density }(\text{cells}/\text{mL})\;=\;\frac{\left(\text{Average live cells }\times \text{ dilution factor}\right)}{\text{Volume of quadrant }(\text{mL})}$$

(2) Total cells = cell density × volume of total culture

### MEV treatment

P8 cells were plated onto 6-well culture plates (Sarstedt, Nümbrecht, Germany: 83.3920) at 2 × 10^5^ cells/well (n = 5 independent wells/treatment). P8 HMC3 cells have been used in previous in vitro studies and were reported to have stable transcriptional output.^20^

**Fig. 9:**
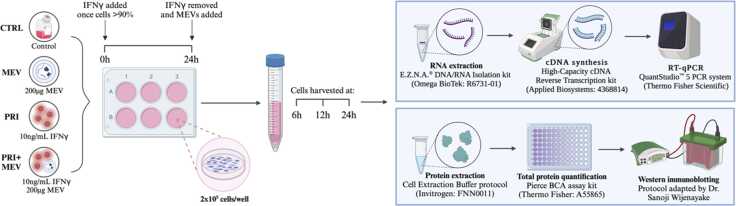
Schematic of priming, supplementation, harvesting, and molecular analysis. Human microglia clone 3 cells were plated at 2×10^5^ cells/well. At >90% confluence, a subset of cells was treated with 10 ng/mL IFN-γ (PRI and PRI+MEV groups). 24 h post-IFN-γ treatment, a second subset of cells was supplemented with 200 µg MEV (MEV and PRI+MEV groups). All cells were harvested at 6 h, 12 h, and 24 h. Total soluble RNA was extracted, and 3000 ng were reverse transcribed into cDNA. RT-qPCR was used to analyze transcript abundance of candidate heat shock response genes. Total soluble proteins were extracted, quantified using BCA assay, and converted to lysate using 2x SDS. Western immunoblotting was used to analyze protein abundance of candidate heat shock proteins. Created in BioRender. Wijenayake, S. (2025) https://BioRender.com/ su78mz0.

A subset of cells was treated with 10 ng/mL IFN-γ (Millipore-Sigma, Burlington, Massachusetts, USA: SRP3058) diluted in Eagle’s Minimum Essential Media with 10% EV-depleted FBS and incubated for 24 h (PRI and PRI + MEV cells). Exactly 10 ng/mL IFN-γ was selected to polarize microglia as per Franck *et al.*^73^ and Peudenier *et al.*^105^ After 24 h IFN-γ incubation, a second subset of cells was supplemented with 200 µg MEVs (MEV and PRI + MEV cells). All cells (CTRL, MEV, PRI and PRI + MEV) were harvested at 6 h, 12 h, and 24 h post-MEV treatment (Figure 9). The 200 µg MEV dosage was selected based on published literature and has been shown to induce molecular responses in human and rodent cell lines.^20^, ^106^

EV-depleted FBS was used for MEV treatment experiments to minimize the biological influence of FBS EVs in the experiment, which may confound MEV effects.^107^ FBS was ultracentrifuged at 100,000× g for 18 h at 4 °C (Beckman-Coulter XL-100; SW55TI Swing-Bucket Rotor; no brake) to pellet FBS EVs. The pellet was discarded, and the supernatant was filtered through a 0.22 µm syringe filter (UltiDent: 229747) prior to use in complete media.

### Cell viability and mitochondrial output

A CyQUANT MTT Cell Viability Assay kit (Thermo Fisher Scientific: V-13154) was used to quantify the mitochondrial output of HMC3 with IFN-γ treatment and MEV treatment as per the manufacturer’s instructions. Absorbance readings were obtained at 570 nm using a BioTek Synergy H1 Multimode microplate reader. Cells were treated with 10 ng/mL IFN-γ for 24 h and/or 200 µg MEVs, with untreated HMC3 cells used as a baseline control, and harvested at 6 h, 12 h and 24 h, as previously described. Based on previous experiments by Franck *et al*.,^73^ 2.5 × 10^4^ cells/well was selected as the optimal plating density and was used for MTT cell viability assays (n = 5 independent wells/treatment). Cell viability was calculated by normalizing blank-adjusted absorbance values to the average of the control cells.^108^, ^109^

### RNA extractions and complementary DNA synthesis

Total soluble RNA (≥18 nucleotides) was extracted from HMC3 cells using the E.Z.N.A.® DNA/RNA Isolation kit (Omega BioTek, Norcross, Georgia, USA: R6731-01) according to the manufacturer’s instructions. RNA concentration (ng/µL) and purity (A260:280 and A260:230 ratios) were measured using a NanoDrop One/One^C^ Microvolume-UV/Vis spectrophotometer (Thermo Fisher Scientific: ND-ONE-W). Samples with A260:A280 ratios of 1.8–2.0 were used for complementary DNA (cDNA) synthesis. An RNA Clean & Concentrator kit (Zymo Research, Irvine, California, USA : R1017) was used to clean select RNA samples with A260:280 ratios <1.8–2.0, according to the manufacturer’s instructions. RNA stability and integrity (Figures S2–S4) were verified by resolving samples on a 1% Tris-acetate-EDTA agarose gel with 2× RNA loading dye (1:1, v/v) (Thermo Fisher Scientific: R0641) stained with Red Safe dye (FroggaBio, Concord, Ontraio, CA: 21141) using a Sub-Cell GT Agarose Gel Electrophoresis System (BioRad:1704401).

Exactly 3000 ng of total soluble RNA was reverse transcribed into cDNA using the High-Capacity cDNA Reverse Transcription kit (Thermo Fisher Scientific: 4368814) according to the manufacturer’s instructions. A T100™ Thermal Cycler (Bio Rad) was used for synthesis using the following amplification parameters: 25 °C for 10 mins; 37 °C for 120 mins; 85 °C for 5 mins; hold at 4 °C. RNA and cDNA were stored at −80 °C.

### Quantitative reverse transcription polymerase chain reaction

mRNA-specific primers were designed using nucleotide sequence information available at the National Center for Biotechnology Information: http://www.ncbi.nlm.nih.gov or obtained from previously published literature. Primer pairs were assessed for compliance with the Minimum Information for Publication of Quantitative Real-Time PCR Experiments guidelines^110^ using sequence information available on National Center for Biotechnology Information as well as using the OligoAnalyzer™ Tool from Integrated DNA Technologies (IDT, Coralville, Iowa, USA). Primer parameters are listed in Table S3. Primer bioinformatics are listed in Table S4.

Quantitative reverse transcription polymerase chain reaction was performed using a QuantStudio™ 5 PCR system (Thermo Fisher Scientific: 96-well and 0.2 mL block) using the Fast SYBR™ Green Master Mix chemistry (Thermo Fisher Scientific: 4385612). Annealing temperatures for primer pairs were determined by testing a range of temperatures approximately ±5 °C of the melting temperature of the forward primer (5′-3′), using the Veriflex setting of the QuantStudio™ 5 PCR system. Temperature testing was conducted using a pool sample (10 ng/µL) representing all test samples. Melt curve analysis was conducted to determine the specificity of the primer pairs. Primer pairs that generated a single, sharp peak void of primer dimers and a derivative reporter >200,000 were used for quantification. A 9-point standard curve ranging from 1000 ng/µL to 3.91 ng/µL was used to determine the optimal cDNA amount to load per primer pair. Analyses of all samples were done in triplicate, and an inter-plate converter was used on all plates to account for variability between plates and runs. Absolute transcript abundance of HSF1 and candidate HSPs (Hsp70, Hsp90, Hsp40, Hsp27) was analyzed using quantity means. The internal controls glyceraldehyde-3-phosphate dehydrogenase (GAPDH), pyruvate kinase (PKM), and 18S were selected as reference genes. Normfinder software (RRID: SCR_003387)^111^ was used to confirm the thermal stability and intragroup/intergroup variability of the reference genes in response to IFN-γ treatment and MEV treatment. All targets were normalized against the geometric mean of the three reference genes.^112^

### Total and cyto-nuclear protein extractions

Total soluble protein was extracted from frozen HMC3 pellets using cell extraction buffer with PIC and phenylmethylsulfonyl fluoride according to the same protocols used for MEV protein extractions. Cyto-nuclear proteins were extracted using the NE-PER™ Nuclear and Cytoplasmic Extraction kit (Thermo Fisher Scientific: 78833) according to the manufacturer’s instructions. Protein concentrations were quantified using a BCA assay. Absorbance readings were obtained at 562 nm. Lysates were solubilized using bromophenol blue loading dye with SDS and *β-*mercaptoethanol, followed by mechanical denaturation (vortexing and temperature). Lysates were stored at −20 °C.

### Western immunoblotting

Exactly 6–15% SDS-polyacrylamide gel electrophoresis was used to resolve protein lysates according to target molecular weights. A 7-point standard curve using protein aliquots from all test samples (5–40 μg) was used to determine the amount of protein to load per target. BLUelf pre-stained protein ladder (FroggaBio: PM008-0500) was used as the molecular weight standard. After determining the optimal loading amount, samples were run with n = 5 independent wells/treatment/timepoint. SDS-polyacrylamide gel electrophoresis was conducted for 60–80 min in 1X Tris-Glycine running buffer (0.3%, w/v Tris-base; 14.4%, w/v glycine; 1%, w/v SDS) at 180 V in a Sub-Cell GT Electrophoresis Cell (BioRad: 1704401). Proteins were transferred from the gel onto a 0.45 µm polyvinylidene fluoride (PVDF) membrane (BioRad: 1620174) using a Trans-Blot Turbo Transfer System (BioRad: 1704150). PVDF membranes were blocked using 1–10% casein in tris buffered saline with tween (30 min, 22 °C) on a rocker and incubated with primary antibody (1:1000, v/v) followed by goat HRP-conjugated anti-rabbit IgG secondary antibody (1:5000, v/v or 1:10,000, v/v) on a rocker (45 min, 22 °C). Blots were visualized using western ECL substrate solution (Thermo Fisher Scientific: 34580) and a ChemiDoc XRS+ chemiluminescence imaging platform (BioRad, Hercules, California, USA) with Image Lab Software version 6.1 (BioRad). The immunoblots were stained with Coomassie brilliant blue (Bioshop: CBB555.10) solution (0.25%, w/v Coomassie blue salt; 7.5% acetic acid; 50%, v/v methanol) for 2 min and destained with destain solution (25%, v/v methanol; 10%, v/v acetic acid; ddH_2_O) for 5 min at room temperature. Coomassie-stained immunoblots were used for total protein normalization, where the intensity of the target ECL bands was normalized against a set of proteins with constant abundance, not including the target protein. ImageJ version 1.5.3 (National Institutes of Health, Bethesda, Maryland, USA) was used to quantify protein abundance.^113^ Western immunoblotting parameters for HSR targets are listed in Table S5. Antibody information for HSR targets is listed in Table S6. ECL and Coomassie images for HSF1 and HSP targets are displayed in Figures S5–S9.

### Statistical analysis

Statistical analysis was carried out using SPSS version 29.0.2.0 (IBM Corp, Armonk, New York, USA), and figures were constructed using GraphPad Prism version 9.5.1 (GraphPad, San Diego, California, USA) and BioRender.com (Toronto, Ontario, CA). Shapiro–Wilk tests were used to test for normality, and Levene’s tests were used to assess homogeneity of variance. All data were normally distributed (*P* > 0.05) and independent by design. As such, parametric testing was used. A two-tailed independent sample t-test was used to determine significant differences across homeostatic or polarized microglia with MEV treatment (*P* ≤ 0.05). One-way analysis of variance was used to determine significant differences across treatment groups for the cyto-nuclear HSF1 data (*P* ≤ 0.05). Univariate general linear model was used to test differences across treatment groups for the MTT cell viability assay (*P* ≤ 0.05) to determine the main effect of priming, the main effect of MEV treatment, and their associations. Extreme outliers with an interquartile range >3 were identified using the SPSS boxplot outlier function and removed from the dataset, only when necessary. No more than one outlier was removed per treatment group. Pairwise comparisons between treatment groups were determined using Tukey HSD post-hoc (*P* ≤ 0.05).

## Funding and support

This research was supported by a Natural Sciences and Engineering Research Council of Canada Discovery grant (RGPIN-2022-03805), and Manitoba Medical Service Foundation Operating Grant (#2021-18) awarded to Dr Sanoji Wijenayake. Jasmyne A. Storm held a Canada Graduate Scholarship from the Natural Sciences and Engineering Research Council of Canada.

## Author contributions

Sanoji Wijenayake: Writing – review & editing, Writing – original draft, Supervision, Funding acquisition, Formal analysis, Conceptualization. Mon Francis Obtial: Data curation. Jueqin Lu: Data curation. Jasmyne A. Storm: Writing – review & editing, Writing – original draft, Validation, Investigation, Formal analysis, Data curation.

## Declarations of interest

The authors declare that they have no known competing financial interests or personal relationships that could have appeared to influence the work reported in this paper.

## Data Availability

Data will be made available on request.
